# Broad-Spectrum Preclinical Antitumor Activity of Chrysin: Current Trends and Future Perspectives

**DOI:** 10.3390/biom10101374

**Published:** 2020-09-27

**Authors:** Ebrahim Rahmani Moghadam, Hui Li Ang, Sholeh Etehad Asnaf, Amirhossein Zabolian, Hossein Saleki, Mohammad Yavari, Hossein Esmaeili, Ali Zarrabi, Milad Ashrafizadeh, Alan Prem Kumar

**Affiliations:** 1 Department of Anatomical Sciences, School of Medicine, Shiraz University of Medical Sciences, Shiraz 7134814336, Iran; e_rahmani@sums.ac.ir; 2 Cancer Science Institute of Singapore and Department of Pharmacology, Yong Loo Lin School of Medicine, National University of Singapore, Singapore 117599, Singapore; e0336095@u.nus.edu; 3 Department of Cell and Molecular Biology, Faculty of Biological Sciences, North Tehran Branch, IslamicAzad University, Tehran 165115331, Iran; etehadsholeh@gmail.com; 4 Young Researchers and Elite Club, Tehran Medical Sciences, Islamic Azad University, Tehran 1916893813, Iran; zabolianamirhossein@gmail.com (A.Z.); hosseinsaleki2015@gmail.com (H.S.); Hosseines@Dr.com (H.E.); 5 Nursing and Midwifery Department, Islamic Azad University, Tehran Medical Sciences Branch, Tehran 1916893813, Iran; mohammadyavari560@gmail.com; 6 Sabanci University Nanotechnology Research and Application Center (SUNUM), Tuzla, Istanbul 34956, Turkey; 7 Faculty of Veterinary Medicine, University of Tabriz, Tabriz 5166616471, Iran

**Keywords:** chrysin, cancer therapy, nanoparticle, flavonoid, chemotherapy

## Abstract

Pharmacological profile of phytochemicals has attracted much attention to their use in disease therapy. Since cancer is a major problem for public health with high mortality and morbidity worldwide, experiments have focused on revealing the anti-tumor activity of natural products. Flavonoids comprise a large family of natural products with different categories. Chrysin is a hydroxylated flavonoid belonging to the flavone category. Chrysin has demonstrated great potential in treating different disorders, due to possessing biological and therapeutic activities, such as antioxidant, anti-inflammatory, hepatoprotective, neuroprotective, etc. Over recent years, the anti-tumor activity of chrysin has been investigated, and in the present review, we provide a mechanistic discussion of the inhibitory effect of chrysin on proliferation and invasion of different cancer cells. Molecular pathways, such as Notch1, microRNAs, signal transducer and activator of transcription 3 (STAT3), nuclear factor-kappaB (NF-κB), PI3K/Akt, MAPK, etc., as targets of chrysin are discussed. The efficiency of chrysin in promoting anti-tumor activity of chemotherapeutic agents and suppressing drug resistance is described. Moreover, poor bioavailability, as one of the drawbacks of chrysin, is improved using various nanocarriers, such as micelles, polymeric nanoparticles, etc. This updated review will provide a direction for further studies in evaluating the anti-tumor activity of chrysin.

## 1. Introduction

Average living standards and access to sufficient healthcare have led to an increase in life expectancy in most regions of the world [1,2]. Although communicable disease-related deaths have been reduced as a result of medical improvements, we have witnessed a 40% increase in cancer-related deaths in recent years. It seems that the number of patients with cancer will increase in the future, and there will be up to 13 million cancer-related deaths by 2030. There are different problems in providing effective cancer therapy, such as the insufficiency of currently applied treatments, lack of early diagnosis, and poor understanding of signaling networks involved in cancer malignancy. In spite of significant attempts in knowing factors contributing to cancer progression, there is not still an effective treatment for cancer [3,4,5]. This is due to the fact that each cancer type has its own features; for instance, cancer cells are different in terms of proliferation, metastasis, and dependence on molecular pathways. Furthermore, cancer cells can obtain resistance to currently applied chemotherapeutic agents [6,7]. Therefore, a novel agent capable of suppressing cancer growth and metastasis and preventing drug resistance is important. In the present review, we aim to reveal the anti-tumor activity of chrysin, as a naturally occurring compound against different cancers. We discuss the various molecular pathways that are affected by chrysin in cancer to direct further studies for investigating more signaling networks. In addition, we describe the role of chrysin in overcoming drug resistance in cancer therapy, which is a major problem in the clinic. Finally, we provide strategies in promoting the anti-tumor activity of chrysin using nanoparticles to enhance bioavailability and therapeutic effects of chrysin.

## 2. Role of Natural Products in Cancer Therapy

Nature is a rich source of compounds with different pharmacological activities [8,9,10,11,12]. The special view towards nature is due to the presence of anti-tumor agents with low toxicity, and capable of suppressing a wide variety of cancers [13,14,15,16,17,18]. Furthermore, natural products are more affordable compared to synthetic drugs. It seems that newly introduced anti-tumor drugs have high similarity to natural anti-tumor compounds. Therefore, identifying novel phytochemicals, making changes in their structure to promote their therapeutic effect, and introducing into the market can be considered as a new way in effective cancer therapy. Newly published experiments have clearly demonstrated the potential of phytochemicals in cancer therapy. The proliferation of cancer cells is suppressed upon the administration of natural anti-tumor compounds [19,20]. Apoptosis and cell cycle arrest can be induced via p53 up-regulation [21]. Based on the fact that poor bioavailability is one of the drawbacks of natural products, using nanoscale delivery systems can exponentially promote their anti-tumor activity against cancer cells for both in vitro and in vivo experiments [22,23,24]. In cancer cells, checkpoint gene expression enhances that provides uncontrolled growth. It has been reported that the administration of natural products is correlated with a decrease in checkpoint expression, and subsequent decrease in proliferation of cancer cells [25]. DNA damage, as well as the activation of both intrinsic and extrinsic pathways of apoptosis, occur during natural product administration in cancer therapy [26]. It is worth mentioning that naturally occurring compounds can promote the efficiency of chemotherapeutic agents in cancer therapy [27,28,29]. For instance, quercetin sensitizes prostate cancer cells to paclitaxel chemotherapy by enhancing reactive oxygen species (ROS) production, stimulation of endoplasmic reticulum (ER) stress, and activation of apoptosis [30]. Molecular pathways. such as MAPK and JNK, are regulated by natural products in apoptosis induction [31]. In addition to proliferation, migration, and invasion of cancer cells can be negatively targeted by natural products [32,33,34]. Increasing evidence confirms the role of epithelial-to-mesenchymal transition (EMT) in cancer metastasis [35,36,37]. Natural products are capable of suppressing the migration of cancer cells by EMT inhibition via down-regulation of upstream molecular pathways, such as Snail and STAT3 [38,39,40].

Taking everything into account, studies agree with the fact that natural products are versatile compounds in cancer therapy, and due to their capacity in targeting various molecular pathways in cancer therapy [41,42,43,44,45], they can be considered as potential agents in the field of cancer therapy. In the next sections, we focus on chrysin as an efficient anti-tumor agent in different cancers.

## 3. Chrysin: An Overview of Chemistry, Sources, and Pharmacokinetics

**Figure 1:**
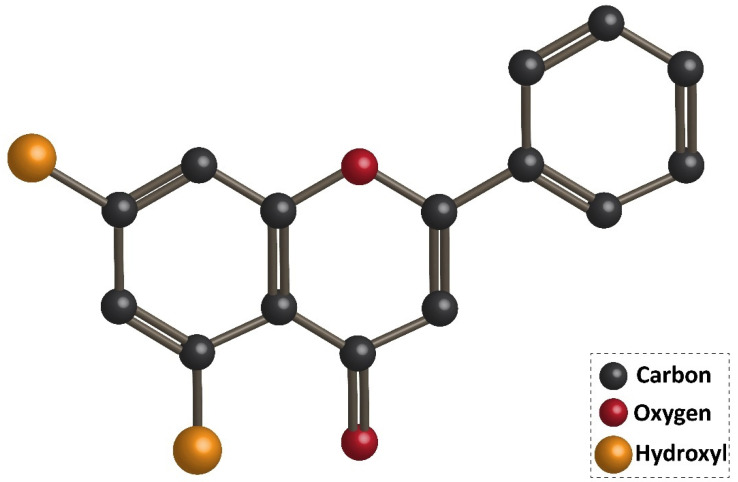
Chemical structure of chrysin.

Flavonoids are the largest group of plant secondary metabolites with favorable health-promoting effects [46,47,48,49]. The interest in flavonoids has been increased, since these valuable compounds act through various physiological mechanisms and affect a wide variety of signaling networks. Dietary intake of flavonoids is estimated to be 50 and 800 mg per day [50,51]. Chrysin is a hydroxylated flavonoid belonging to flavone class, and is extensively found in sources, such as honey, propolis, and plant species [52,53]. Noteworthy, chrysin occurs in natural sources with different concentrations. For instance, the concentration of chrysin in honeydew honey is 0.10 mg/kg, while it has a higher concentration (5.3 mg/kg) in forest honeys [54]. The content of chrysin in propolis is estimated to be 25 g/L [55]. Chrysin concentration in mushrooms is at the range of 0.17–0.34 mg/kg [56]. The IUPAC name of chrysin is 5,7-dihydroxy-2-phenyl-4H-chromen4-one and 5,7-dihydroxyflavone. Figure 1 demonstrates the chemical structure of chrysin. The chrysin structure has similarities and differences with the flavonoid family. Structurally, chrysin has two benzene rings (A and B) with one oxygen consisting of a heterocyclic ring. Chrysin lacks a 3-carbon hydroxyl group, but it has 2–3 double-bound carbon with a carbonyl group attached to 4th carbon. The chemical structure of chrysin demonstrates that it has –OH group at 5th and 7th carbon atoms. There is a difference in the structure of chrysin and other flavonoids, so that chrysin does not possess any oxygenation in ring B (Figure 1). It has been reported that changes in ring A of chrysin account for the generation of different derivatives of chrysin, such as wogonin, baicalein, and oroxylin [57].

Accumulating data demonstrates that poor absorption, rapid metabolism, and systemic elimination are responsible for poor bioavailability of chrysin in humans that, subsequently, restrict its therapeutic effects [58]. It is worth mentioning that oxidation in intestinal and hepatic cells is not responsible for the metabolism of chrysin in the body. In contrast, conjugation pathways, such as glucuronidation and sulfation catalyze chrysin. Enzymes, such as P-PST, M-PST, and UGT1A6, contribute to the metabolism of chrysin, and their high affinity for chrysin can justify the poor bioavailability of this natural compound. Clinical studies have shown that the plasma concentration of chrysin following oral administration is very low [59]. Notably, serum concentrations of chrysin have not been reported yet, but it can be predicted based on other flavonoids. Since flavonoid aglycones demonstrate serum concentration as low as 1 µmol/L [60], the serum concentration of chrysin would be at the range of nanomolar. The studies related to the absorption of chrysin demonstrate that its sulfation and glucuronidation limit the absorption of this valuable compound in the intestine. MRP2 transporters are involved in the efflux of chrysin metabolites from the intestine, and in the lumen, sulfatases and glucuronidases hydrolyze metabolites into chrysin. This leads to the emergence of chrysin in stool, but high contents of chrysin in stool demonstrates that it has low absorption [61]. Some strategies have been applied in promoting bioavailability and absorption of chrysin, such as using nanoscale delivery systems [62].

## 4. Chrysin and Its Pharmacological Activities

In previous sections, we provided explanations about the role of natural products in cancer therapy, and then, we introduced the chemistry and pharmacokinetics of chrysin. In this section, we aim to describe the pharmacological activities of chrysin, based on the newly published article—which is summarized in Table 1.

**Figure 2:**
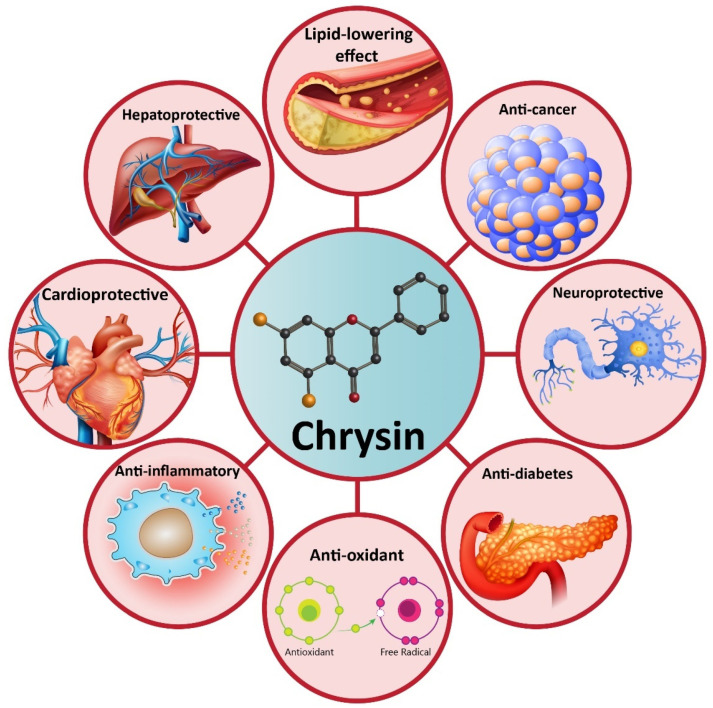
A schematic representation of the health-promoting effects of chrysin in pre-clinical experiments.

Increasing evidence demonstrates that chrysin possesses health-promoting effects, including antioxidant [63,64], anti-inflammatory [65], anti-diabetes [66], neuroprotective [67], hepatoprotective [68], cardioprotective [69], lipid-lowering effect [70], etc. These therapeutic effects have made chrysin as a suitable option in disease therapy. Non-alcoholic fatty liver disease (NAFLD) is one of the most common metabolic disorders, and to date, natural products have shown great potential in the alleviation of NAFLD. Similarly, a recently recorded article has revealed that chrysin administration (25, 50, and 100 mg/kg) alleviates NAFLD in rats via reducing serum fasting glucose that subsequently improves insulin resistance and dyslipidemia. Noteworthy, chrysin can significantly diminish liver weight by reducing hepatic free fatty acids, triglyceride, and cholesterol content. Anti-inflammatory and antioxidant activities of chrysin are also involved in the amelioration of NAFLD via decreasing lobular inflammation, steatosis, and carbonyl content [71]. Many reports demonstrate that chrysin can be beneficial in reducing acetaminophen-mediated hepatotoxicity in rats. In this regard, chrysin reduces levels of pro-inflammatory cytokines, such as tumor necrosis factor-α (TNF-α) and interleukin-2 (IL-2). The ameliorative effect of chrysin on acetaminophen-mediated hepatotoxicity seems to be dose-dependent with more therapeutic effects at higher concentrations [72]. In addition to hepatoprotective activity, chrysin has shown potential neuroprotective effects. One of the complications causing neuronal cell death is ischemic-reperfusion (I/R) injury. Inflammation and oxidative stress are two main mechanisms involved in I/R injury [73,74,75]. Chrysin administration (10 and 20 mg/kg) reduces pro-inflammatory factors (TNF-α, IL-1β, and IL-6) and oxidative stress to alleviate cerebral I/R injury. Investigation of molecular pathways reveals that the induction of the PI3K/Akt signaling pathway by chrysin contributes to a reduction in oxidative stress and inflammation during cerebral I/R injury [76]. The inhibitory effect of chrysin on inflammation and oxidative stress is also important in Parkinson’s disease (PD) treatment [77]. Chrysin (25, 50, and 100 mg/kg) improves cognitive capacity, inflammation, and apoptosis to ameliorate traumatic brain injury (TBI) [78]. Overall, the literature confirms the health-promoting and therapeutic effects of chrysin that are important in disease therapy, and the effect of this valuable compound on molecular pathways (Figure 2) [79,80,81,82]. In the next sections, we specifically discuss the role of chrysin in cancer therapy [83,84].

## 5. Chrysin and Cancer

### 5.1. Breast Cancer

Breast cancer is the most common and malignant cancer in women [94,95,96]. Recurrence and chemoresistance have restricted the efficacy of currently applied treatment in breast cancer therapy [97,98,99,100]. Natural products have demonstrated an excellent inhibitory effect on both proliferation and metastasis of breast cancer [101,102,103,104]. A combination of chrysin and silibinin is beneficial in suppressing breast cancer malignancy via decreasing cancer proliferation. Furthermore, chrysin and silibinin induced cell cycle arrest via down-regulation of cyclin D1 and hTERT [105]. The epidermal growth factor receptor (EGFR) is considered as a potential target in cancer therapy [106]. Standard chemotherapy reduces the replication of cancer cells, but EGFR inhibitors are capable of cancer proliferation and survival [107]. Therefore, using EGFR inhibitors, such as antibody-based immunoconjugates, monoclonal antibodies, antisense oligonucleotides, and small molecules is preferred to chemotherapy [108]. A new derivative of chrysin known as CHM-04 has been synthesized with affinity to EGFR. It seems that CHM-04 is a potent inhibitor of EGFR with more efficiency compared to chemotherapeutic agents in suppressing cancer malignancy. In triple-negative breast cancer cells treated with chrysin, sphere formation ability, proliferation, and migration are substantially suppressed that can be attributed to the inhibitory effect of CHM-04 on EGFR [109].

Low oxygen level is known as hypoxia, and is a common feature of solid tumors. Increasing evidence demonstrates that hypoxia is responsible for the growth and progression of cancer cells, and it is one of the best targets in cancer therapy [110,111,112]. Noteworthy, clinical studies revealed the relationship between hypoxia and cancer progression and metastasis [113,114]. In hypoxia, vascular endothelial growth factor (VEGF) is induced that promotes proliferation and invasion of cancer cells. Furthermore, hypoxia adaptation is mediated by hypoxia-inducible factor-1 (HIF-1) that is an efficient target in cancer therapy. In addition to HIF-1, other molecular pathways, such as signal transducer and activator of transcription 3 (STAT3), play a key role in hypoxia-mediated VEGF gene expression [115,116,117,118]. Administration of chrysin is associated with the disruption of hypoxia-induced VEGF gene expression. Moreover, chrysin is capable of reducing STAT3 phosphorylation in hypoxic conditions without affecting the HIF-1α protein level. In vitro and in vivo experiments agree with the fact that chrysin is a potent agent in suppressing metastasis and proliferation of breast cancer cells during hypoxic conditions, since chrysin abrogated lung metastasis of breast cancer cells [119].

Increasing evidence demonstrates that combination therapy is of interest in promoting the anti-tumor activity of agents. Although chrysin has demonstrated great potential in suppressing proliferation and metastasis of cancer cells, its anti-tumor activity can be promoted by combination therapy. Metformin, as an anti-diabetic agent, has been applied in cancer therapy, due to its capacity in inhibiting proliferation, metastasis, and induction of apoptosis, and cell cycle arrest [120,121]. It seems that combination therapy of breast cancer cells using chrysin and metformin exerts a synergistic effect and is more efficient compared to chrysin alone. Cyclin D1 and hTERT are down-regulated by chrysin and metformin in breast cancer therapy [122].

### 5.2. Lung Cancer

International Agency for Research on Cancer has considered nickel as one of the carcinogenic agents [123,124,125]. Exposing to nickel-containing compounds is correlated with the risk of lung cancer development [126,127]. Enhancing ROS levels, inflammation induction, epigenetic gene regulation, and stimulation of signaling pathways are positively affected by nickel in cancer development [128,129,130]. Furthermore, activation of toll-like receptors (TLRs) is associated with cancer development [131,132]. The nuclear factor-kappaB (NF-κB) signaling pathway promotes inflammation and cancer progression [133]. A report has evaluated and compared the efficiency of five natural products, including quercetin, chrysin, curcumin, apigenin, and luteolin. Among them, quercetin and chrysin demonstrated the highest efficacy in lung cancer treatment. A combination of quercetin and chrysin reduced levels of pro-inflammatory factors, such as IL-1β, Il-6, TNF-α, and IL-10, via NF-κB down-regulation. Furthermore, chrysin and quercetin decreased expressions of Myd88 and TLR4, as well as MMP-9, to suppress the viability and metastasis of lung cancer cells [134].

### 5.3. Prostate Cancer

Prostate cancer (PC) is one of the most common cancers in men that is responsible for 21% of cancer cases and 8% of cancer-related deaths in the United States [135,136,137]. Chemotherapy, radiotherapy, and prostatectomy are strategies in PC therapy, but recurrence and resistance of PC cells are problems, requiring novel strategies in PC therapy [138,139]. Increasing evidence demonstrates that PI3K/Akt and MAPK signaling pathways account for an increase in proliferation and metastasis of cancer cells, and their inhibition is important in cancer therapy [140,141,142,143]. In PC cells, chrysin down-regulates the expression of the PI3K/Akt pathway to interrupt the proliferation of PC cells. Furthermore, MAPK down-regulation by chrysin leads to a decrease in PC proliferation. Chrysin is able to induce apoptosis in PC cells via mitochondrial dysfunction, so that after chrysin administration, an increase occurs in levels of ROS that, subsequently, impairs the integrity of the mitochondrial membrane, leading to cytochrome C release and apoptosis induction [144]. Noteworthy, in addition to mitochondria, ER can also participate in apoptosis. The primary role of ER is to preserve cell homeostasis and ensuring the correct conformation of proteins. ER stress occurs when levels of unfolded proteins exceed from the capacity of ER. This leads to the activation of unfolded protein response (UPR) that, subsequently, stimulates PRKR-like ER kinase (PERK), eukaryotic translation initiation factor 2α (eIF2α), and 78 kDa glucose-regulated protein (GRP78) [145,146,147,148]. Chrysin administration also impairs ER homeostasis to induce ER-mediated apoptosis in PC cells [144].

### 5.4. Ovarian Cancer

Ovarian cancer (OC) is the fifth leading cause of death in women, and is considered one of the most lethal gynecologic cancers [135,149]. Based on the experiments performed in the field of OC treatment, it seems that phytochemicals are potential therapeutic agents in this case [150,151]. In the previous section, we discussed that mitochondrial dysfunction leads to apoptosis induction [152]. Upon chrysin administration, an increase occurs in levels of ROS and cytoplasmic Ca^2+^ that mediate apoptosis induction in OC cells [153]. However, this study provides controversial results about the role of molecular pathways that needs to be explored in further studies. Accumulating data demonstrates that the PI3K/Akt signaling pathway contributes to cancer proliferation and metastasis. PI3K/Akt inhibition has been suggested in different experiments as a promising strategy in cancer therapy [154,155,156]. However, a previous study has shown that chrysin suppresses OC malignancy via PI3K/Akt and MAPK induction [153]. Therefore, further studies are required to shed some light on this area.

### 5.5. Gastric Cancer

Gastric cancer (GC) is the third leading cause of cancer death, with 783,000 deaths in 2018 [157,158,159,160]. Different factors are involved in GC progression, and ten-eleven translocation (TET) enzyme is one of them. TET enzymes contribute to the oxidation of 5-methylcytosine (5mC) to 5-hydroxymethylcytosine (5hmC) and participate in epigenetic modification [161]. Studies show the role of TET enzymes in GC development. For instance, TET1-mediated demethylation stimulates the aggressive behavior of GC cells [162]. TET2 exerts RASSF1A methylation to affect malignant cell activity [163]. Furthermore, down-regulation of TET3 has been shown in GC [164]. The effect of chrysin on GC cells has been investigated in vitro and in vivo. In MKN45 cells, chrysin promotes the expression of TET1 and 5hmC to stimulate apoptosis and disrupt migration and invasion of GC cells. Furthermore, TET1 deletion by CRISPR/Cas9 system in a mouse model leads to the development of GC, and chrysin administration can be considered as a promising strategy in GC suppression [165].

One of the properties of phytochemicals is their capability to regulate microRNA (miR) expression [166,167]. Briefly, miRs are non-coding parts of the genome that are not transcribed into protein [168]. Cellular mechanisms, such as proliferation, migration, differentiation, etc., are tightly regulated by miRs [169]. Disturbance in miR expression leads to the emergence of pathological conditions, particularly cancer [170,171]. Chrysin is capable of promoting the expression of miR-9 and Let-7a as onco-suppressor factors in cancer to inhibit the proliferation of GC cells. Using nanoparticles can significantly promote the ability of chrysin in enhancing miR-9 expression [172].

### 5.6. Cervical Cancer

Cervical cancer is one of the most common malignancies diagnosed in women [173,174,175]. Chronic infection with high-risk human papillomavirus and inherited polymorphism of cytokine genes are involved in cervical cancer emergence [176,177,178,179,180]. Hence, enhanced levels of cytokines participate in cervical cancer progression. Furthermore, EMT-related metastasis provides a poor prognosis of patients with cervical cancer [181]. Hence, anti-tumor compounds with a modulatory effect on inflammation can be beneficial in suppressing cervical cancer metastasis. Exposing cervical cancer cells into transforming growth factor-beta (TGF-β) is associated with enhanced levels of TNF-α, inflammation, and metastasis. As a consequence of inflammation, NF-κB is activated that induces Twist/EMT axis in cervical cancer metastasis. Chrysin (5, 10 and 20 µM) suppresses the aggressive behavior of cervical cancer cells in a dose-dependent manner. Down-regulation of NF-κB, and subsequent decrease in Twist/EMT are mediated by chrysin administration, negatively affecting cervical cancer metastasis [182].

Scutellaria discolor Colebr is a well-known medicinal plant species with therapeutic effects in treating different diseases [183]. There have been efforts in revealing bioactive compounds in this plant that are responsible for its pharmacological activities, particularly cancer. It has been reported that chrysin is the major bioactive component of this plant that provides the anti-tumor activity against cervical cancer cells. Induction of cell cycle arrest and apoptosis via up-regulation of caspase-3, caspase-9, and Bax are mediated by chrysin. Moreover, chrysin impairs the proper function of mitochondria via providing mitochondrial membrane depolarization, leading to reduced viability of cervical cancer cells [184].

### 5.7. Liver Cancer

Studies are in line with the fact that cancer cells are different from normal cells in terms of metabolism [185]. Aerobic glycolysis, or the Warburg effect, is one of the hallmarks of cancer that was first recognized in 1920 by Otto Heinrich Warburg [186]. In this process, regardless of oxygen levels, glucose is converted into lactate to meet the needs of cancer cells into energy, leading to their uncontrolled proliferation [187]. Different factors have been recognized to participate in changing the metabolism of cancer cells from the Krebs cycle to glycolysis, and hexokinases (HKs) are one of them [188,189]. A large body of evidence shows the important role of HK-2 in the Warburg effect in different cancers [190,191,192,193]. Chrysin administration (15, 30, and 60 mM) reduces the expression of HK-2 in hepatocellular carcinoma (HCC) cells to impair glucose uptake and lactate production. In addition to glycolysis metabolism impairment, the inhibitory effect of chrysin on HK-2 leads to apoptosis, so that chrysin disrupts the interaction of HK-2 and VDAC-1 on mitochondria that releases Bax from mitochondrial into the cytoplasm, leading to apoptosis induction. Notably, tumor xenografts treated with chrysin demonstrated a decrease in HK-2 levels in tissues [194].

The main pathway that is followed by chrysin in suppressing liver cancer survival is apoptosis induction. In this way, chrysin substantially enhances levels of ROS that, subsequently, disturbs mitochondrial function. Disruption in the integrity of the mitochondrial membrane leads to cytochrome C release into the cytoplasm, resulting in apoptotic cell death [195].

Increasing evidence is in agreement with the fact that the STAT3 signaling pathway participates in the proliferation and invasion of HCC cells [196,197,198,199]. Inhibition of STAT3 by anti-cancer agents is important in effective HCC therapy [200,201,202]. In HCC cells exposed to chrysin, a decrease occurs in sphere formation capacity. Investigation of molecular pathways reveals that STAT3 undergoes down-regulation upon chrysin administration. Notably, an upstream modulator of STAT3 known as SHP-1 is up-regulated by chrysin, and consequently, it decreases expression of STAT3, leading to inhibited sphere formation [203].

### 5.8. Melanoma

Melanoma is a highly resistant and malignant tumor of the skin that is responsible for about 3% of all cancer cases. Over the past decades, we have witnessed an increase in the occurrence of melanoma. Although melanoma accounts for 4% of all skin cancer cases, its aggressiveness and malignancy have led to comprising 80% of all deaths from skin cancer [204]. Melanoma, at the first stages, can be treated with surgery, but in an advanced stage, it metastasizes into other sites, making its treatment more complex [205,206,207]. Plant derived-natural compounds can be considered as potential agents in melanoma therapy, due to their ability in apoptosis and cell cycle induction, and inhibiting migration [208,209,210]. Chrysin is a potent agent in melanoma therapy, and this ability has been approved in vitro and in vivo. Chrysin stimulates apoptosis and cell cycle arrest (G2/M phase) in a dose-dependent manner. In tumor xenografts, chrysin decreases tumor growth by 60% after 14 days of treatment, while this number enhances to 70% after 21 days of treatment. Noteworthy, in melanoma therapy, chrysin promotes cytotoxicity activity of natural killer cells, macrophages, and cytotoxic T cells [211].

MMPs are involved in enhancing the invasion of cancer cells via extracellular matrix (ECM) degradation [212,213]. MMP-2 and MMP-9 provide metastasis of cancer cells into distant organs via degrading matrix collagen and basement membrane [214,215]. Chrysin (5–15 µM) suppresses metastasis of melanoma cells via down-regulation of MMP-2. Furthermore, N-cadherin and E-cadherin are respectively down-regulated and up-regulated upon chrysin administration in inhibiting melanoma invasion [182]. In previous sections, we discussed the oncogene role of NF-κB and PI3K/Akt signaling pathways in cancer. Chrysin treatment is associated with a decrease in expression of NF-κB and PI3K/Akt to suppress melanoma proliferation [182].

### 5.9. Bladder Cancer

The second most common type of tract cancer in developed countries is bladder cancer. Its incidence rate is around 400,000 cases, with approximately 160,000 death annually [135]. Chemotherapy is not suggested in bladder cancer therapy, due to side effects and chemoresistance [216]. Novel strategies can be developed for promoting the efficacy of chemotherapy in bladder cancer therapy, such as using phytochemicals with anti-tumor activity [217,218]. On the other hand, molecular pathways, such as STAT3 participate in bladder cancer progression [219]. STAT3 can individually promote the proliferation of bladder cancer cells [220], or it may be targeted by upstream mediators, such as Akt/ERK [221]. Administration of chrysin is correlated with an increase in ROS levels to down-regulate STAT3 expression. Furthermore, chrysin activates the intrinsic pathway of apoptosis via caspase-3 and caspase-9 up-regulation. Anti-apoptotic factors, such as Bcl-2, Mcl-1, and Bcl-xl undergo down-regulation by chrysin in bladder cancer cells. Notably, chrysin substantially diminishes survival by ER stress induction via stimulating UPR, PERK, ATF4, and elF2α [222].

### 5.10. Colorectal Cancer

Colorectal cancer (CRC) is a heterogeneous disease with a rise in the incidence rate in recent years. Both molecular and pathological properties determine the prognosis and response of CRC cells into therapy [223,224]. 5-fluorouracil (5-FU) is extensively applied in treating patients with CRC, but drug resistance and side effects have restricted its use [225,226]. Recently, chrysin has been considered as a substitution for 5-FU in CRC therapy. Chrysin administration (5–50 µM) is associated with a significant decrease in the viability of CRC cells [227]. An investigation into the molecular mechanisms demonstrates that autophagy is affected by chrysin in CRC therapy. Autophagy is a “self-digestion” process with stimulation upon stressful conditions, such as ER stress, mitochondrial damage, starvation, etc. [228,229]. Autophagic cell death is important in reducing the viability of cancer cells [230,231]. Chrysin enhances levels of light chain-3 II (LC-3II) to induce autophagy. Furthermore, by promoting ROS generation, chrysin down-regulates the expression of the mammalian target of rapamycin (mTOR) to stimulate autophagy, leading to a decrease in the viability of CRC cells [227].

It is worth mentioning that irradiation can improve the anti-tumor activity of chrysin against colon cancer cells. Irradiation technology is able to promote biological properties or physical features of biomolecules through structural modification [232,233,234]. Recently, chrysin and gamma irradiation have been co-applied in colon cancer therapy. Irradiation substantially enhances the cytotoxic activity of chrysin. This inhibitory effect against colon cancer cells is exerted via promoting ROS generation, inducing mitochondrial dysfunction, activation of a caspase cascade (caspase-3 and caspase-9), and stimulating cleavage of poly (adenosine diphosphate-ribose) polymerase (PARP) [235].

Peroxisome proliferator-activated receptor alpha (PPARα) is a crucial member of the superfamily of nuclear hormone receptors with regulatory effects on migration, proliferation, metabolism, etc. [236,237,238,239]. Increasing evidence demonstrates that using a specific ligand for stimulation of PARPα is of interest in suppressing cancer growth [240,241]. On the other hand, cytochrome P450 (CYPs) enzymes contribute to drug metabolism and are found in different organs of the body, such as lung, liver, etc. [242,243]. PARPα is able to regulate gene expression of CYPs, such as CYP3A4 and CYP2C8 [244]. Chrysin administration significantly enhances the expression of PARPα in cancer cells. This leads to a significant reduction in expression of CYP2S1 and CYP1B1, leading to decreased proliferation (cell cycle arrest) and migration of cancer cells [245].

**Figure 3:**
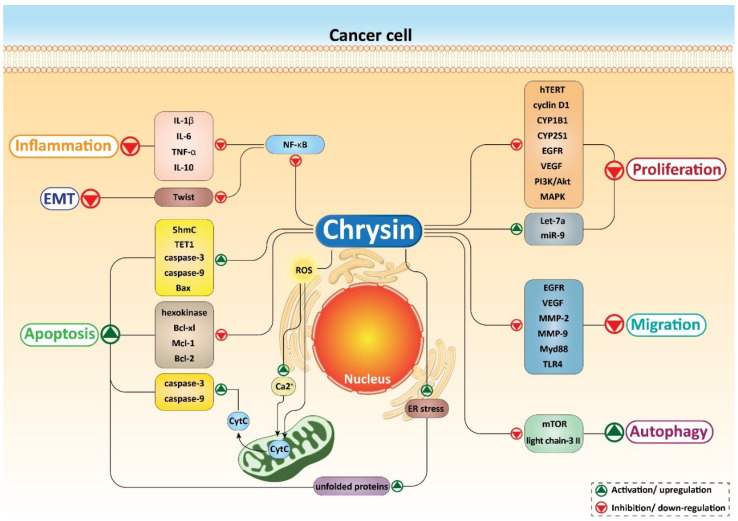
Mechanisms involved in the anti-tumor activity of chrysin against different cancers. IL-1β, interleukin-1β; IL-6, interleukn-6; TNF-α, tumor necrosis factor-α; NF-κB, nuclear factor-kappaB; ROS, reactive oxygen species; ER, endoplasmic reticulum; Mcl-1, myeloid cell leukemia-1; EGFR, epidermal growth factor receptor; VEGF, vascular endothelial growth factor; miR, microRNA; MMP, matrix metalloproteinase; TLR4, toll-like receptor 4; mTOR, mammalian target of rapamycin.

A schematic summary on anti-tumor effects of chrysin in cancer is shown in Figure 3. Table 2 list chrysin administration in treating various cancers.

## 6. Chrysin, Chemotherapy and Drug Resistance

Chemotherapy is an inevitable part of cancer therapy, but its potential has been restricted in recent years, due to the resistance of cancer cells [259,260]. In fact, chemoresistance of cancer cells has urged scientists to seek new anti-tumor agents [261]. Based on the role of natural products in cancer treatment, they can be beneficial in sensitizing cancer cells into chemotherapy [262,263]. That is why these valuable agents have been extensively co-administered with chemotherapeutic agents in cancer therapy. Anti-tumor phytochemicals can suppress proliferation, metastasis, and malignant behavior of cancer cells that are in favor of chemotherapeutic agents [102,264,265]. In this section, we provide a discussion about the role of chrysin as a naturally occurring compound in reversing drug resistance.

Cisplatin is a well-known chemotherapeutic agent with clinical application. However, resistance is the most important reason for treatment failure with this agent in the clinic [266,267]. Various molecular pathways have been suggested to participate in cisplatin resistance, such as CLEC4M, miRs, lncRNAs, etc. [268,269]. In respect to the high anti-tumor activity of chrysin, this plant derived-natural compound can be advantageous in suppressing chemoresistance. Noteworthy, it has been reported that selenium-containing chrysin and quercetin derivatives are potent agents in reversing cisplatin resistance [270].

Docetaxel (DTX) is a commercially applied chemotherapeutic agent in treating lung cancer, breast cancer, gastric cancer, etc. DTX stimulates apoptosis and cell cycle arrest via attaching β-tubulin into microtubules and disrupting cancer growth [271]. Similar to other chemotherapeutic agents, cancer cells are capable of obtaining resistance to DTX [272]. Moreover, the anti-tumor activity of DTX can be improved by combinational therapy [273]. A combination of chrysin (20–100 µM) and DTX is advantageous in suppressing the proliferation of cancer cells, and inducing growth delay in tumor xenografts [274]. This is distributed to apoptosis induction by chrysin that, subsequently, sensitizes cancer cells into DTX chemotherapy [274].

P53 is a key player in apoptosis induction. It stimulates apoptosis in both transcription-dependent and transcription-independent manners. In the transcription-dependent pathway, down-stream genes of p53 are regulated to induce apoptosis in cancer cells [275,276,277]. Furthermore, p53 is capable of moving out of the nucleus, and interacting with mitochondria and its proteins, such as Bcl-2 and Bcl-xl, in apoptosis induction [278]. In liver cancer cells exposed to chrysin and cisplatin, an increase occurs in phosphorylation and accumulation of p53 via ERK1/2 up-regulation. Consequently, apoptotic factors, such as Bax and DR5, undergo up-regulation, while a decrease occurs in the expression of anti-apoptotic factor Bcl-2. The intrinsic pathway of apoptosis is activated via caspase-8 activation. Chrysin and cisplatin also induce the extrinsic pathway of apoptosis via releasing cytochrome C into the cytoplasm and activating caspase-9 [279].

Nuclear factor erythroid 2-related factor 2 (Nrf2) is an important signaling pathway involved in antioxidant activity against oxidative stress and other kinds of stresses [280,281,282]. Recently, much attention has been directed towards the role of Nrf2 in the chemoresistance of cancer cells [283]. Nrf2 follows different routes in exerting chemoresistance, such as enhancing expression of CD99 [284], inhibiting DNA damage [285], and reducing oxidative stress-mediated damage [286]. Therefore, Nrf2 inhibition is important in reducing chemoresistance. Chrysin administration (10 and 20 mM) promotes the sensitivity of cancer cells into doxorubicin chemotherapy. Further analysis reveals that Nrf2 undergoes down-regulation by chrysin in cancer cells. Furthermore, in reducing Nrf2 expression, chrysin down-regulates the expression of ERK and PI3K/Akt pathways—leading to an increase in the efficiency of doxorubicin in chemotherapy [287].

## 7. Chrysin-Loaded Nanoparticles in Cancer Therapy

Micelles have attracted much attention in cancer therapy, due to their potential to deliver anti-tumor agents [288,289]. Self-assembled micelles are amphiphilic copolymers with size at the range of 10–100 nm. Micelles have high cellular uptake and passive targeting functions to tumor known as enhanced permeability [290,291]. Recently, chrysin- and docetaxel-loaded micelles have been applied in enhancing the efficacy of chemotherapy. This co-delivery by micelles exerts a synergistic effect on chemotherapy and effectively suppresses migration and invasion of cancer stem cells. Chrysin- and docetaxel-loaded micelles enhance levels of ROS to impair cancer stem cell viability. Notably, enhanced the anti-tumor activity of chrysin and docetaxel against cancer cells is due to their enhanced accumulation in cancer cells by micelles [292]. Polymeric micelles have also been designed in co-delivery of chrysin and methotrexate in the chemotherapy of breast cancer cells. The idea of using a chemotherapeutic agent with a natural anti-tumor agent is that this combination is important in sensitizing cancer cells into chemotherapy. Using nanoparticles promotes cytotoxicity against cancer cells via enhancing cellular uptake. Based on the small size of polymeric micelles (around 55 nm), they can escape from macrophages and kidney filtration to reach into the tumor site, providing targeted delivery of anti-tumor compounds [293].

Another study has applied polyurea dendrimers for delivery of chrysin in ovarian cancer therapy. Polyurea dendrimers are three-dimensional polymers with urea moieties in the backbone and peripheral amine groups. They possess various beneficial properties, including water-solubility, biocompatibility, biodegradability, and pH-sensitivity, making them suitable options in drug delivery [294]. Furthermore, as cancer cells overexpress folate receptors on their surface [295,296], surface functionalization of nanoparticles with folate can be advantageous in enhancing cellular uptake of these nanoparticles and providing selective targeting. Chrysin- and selenium-loaded dendrimers are capable of induction of oxidative stress and reducing the viability of OC cells. Furthermore, they demonstrate no toxicity against normal cells that can be attributed to using folate for the functionalization of dendrimers [297].

Polymeric nanoparticles possess a core-shell structure that self-assemble in an aqueous medium. The hydrophilic shell is responsible for preserving the stability of nanoparticle, and the hydrophobic core encapsulates anti-tumor drug. Synthetic polymers, including poly (e-caprolactone) (PCL), polyglycolide (PGA), and polylactides (PLA), are applied in biomedical applications, due to their features, such as biocompatibility, high permeability, predictable degradation kinetics, etc., that are important in the field of biomedicine [298,299,300]. However, crystallinity and low biodegradation are drawbacks of PCL that can be solved using monomers. Poly (ethylene glycol) (PEG) is a safe, flexible, and hydrophilic agent approved by the Food and Drug Administration (FDA) that can be used internally in the human body [298,301,302,303]. Chrysin-loaded polymeric nanoparticles have been applied in breast cancer therapy. The results demonstrate that targeted delivery of chrysin at the tumor site by polymeric nanoparticles leads to enhanced anti-tumor activity, due to enhanced cellular uptake [304].

Nanoparticles can provide a platform for co-loading of chrysin with other natural anti-tumor compounds, such as curcumin. Briefly, curcumin is isolated from the rhizome of curcuma longa and has potent anti-tumor activity against different cancer cells [305]. Using nanoparticles can significantly enhance the bioavailability and therapeutic effects of curcumin [306]. Curcumin- and chrysin-loaded PLGA-PEG nanoparticles have been designed in CRC therapy. This co-loading exerts a synergistic effect and enhances the cytotoxicity of these phytochemicals against CRC cells [307]. Studies demonstrate that telomerase activity is associated with enhanced proliferation and invasion of cancer cells. Catalytic domain (hTERT) participates in telomerase gene overexpression that has been reported in CRC [308,309]. Chrysin- and curcumin-loaded nanoparticles effectively down-regulate the expression of hTERT in suppressing the progression of CRC cells [307]. In addition to the anti-proliferative activity via hTERT down-regulation, chrysin- and curcumin-loaded nanoparticles can suppress metastasis of cancer cells via reducing expressions of MMP-2 and MMP-9 [310].

**Figure 4:**
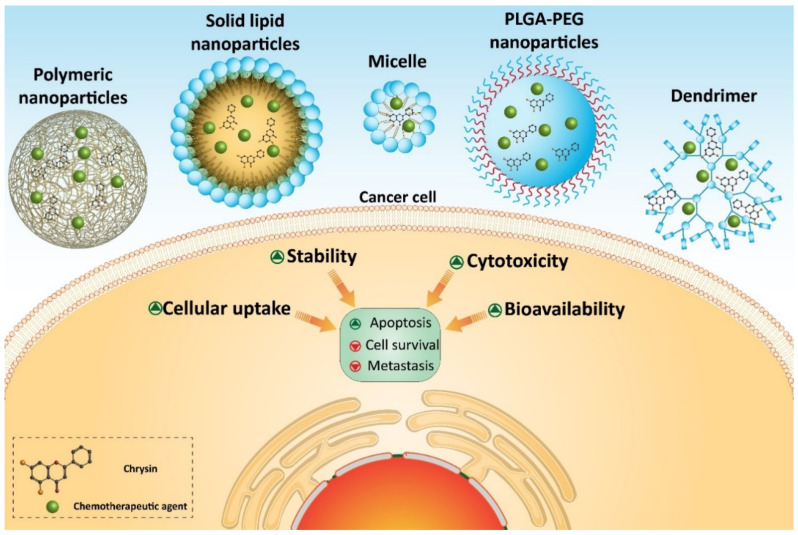
Chrysin-loaded nanoparticles in cancer therapy.

Several homologous proteins known as tissue inhibitors of metalloproteinase (TIMPs) can regulate the activity of MMPs. TIMP-1 and TIMP-2 are capable of reducing the expression of MMP-2 and MMP-9 in suppressing metastasis and migration of cancer cells [311]. Chrysin- and curcumin-loaded nanoparticles significantly promote the expression of TIMP-1 and TIMP-2 to exert a reduction in melanoma invasion [310]. Taking everything into account, studies agree with the fact that nanoparticles can enhance the anti-tumor activity of chrysin against cancer cells [62,312,313,314,315,316]. Nanoparticles can provide a platform for the co-delivery of chrysin and other anti-tumor agents that is important in promoting its inhibitory effect against cancer cells (Figure 4) (Table 3). Further studies can focus on developing other types of nanocarriers, such as carbon nanotubes, liposomes, etc., for delivery of chrysin in cancer therapy.

## 8. Conclusions and Remarks

In the present review, we provided a mechanistic review of chrysin and its underlying mechanisms for anti-tumor activity [323,324,325]. Noteworthy, chrysin derivatives have also shown potential anti-tumor activity [326,327,328,329], showing that future studies can focus on chemical modification of chrysin structure in improving its bioavailability, anti-tumor activity, etc. Although chemical modification is a promising strategy in promoting the anti-tumor activity of chrysin, it seems that nanoscale delivery systems, such as polymeric nanoparticles, liposomes, solid lipid nanoparticles, etc., can also be considered in promoting cellular uptake of chrysin and enhancing its anti-tumor activity.

Chrysin affects various molecular pathways and mechanisms in cancer therapy. Apoptosis is the most well-known target of chrysin in cancer therapy, and both intrinsic and extrinsic pathways of apoptosis are induced by chrysin in cancer cells. Disrupting homeostasis of mitochondria and ER are followed by chrysin in apoptosis induction in cancer cells. Autophagy is another programmed cell death that is activated by chrysin in cancer therapy. As autophagy has a dual role in cancer, meaning it may suppress cancer progression, or may function as a pro-survival factor in promoting the proliferation of cancer cells [330,331,332,333], much attention should be directed towards the regulation of autophagy by chrysin in cancer therapy. It has been reported that chrysin induces autophagy in cancer therapy, showing the anti-tumor role of autophagy. However, more studies will reveal a relationship between chrysin and autophagy in cancer therapy. In terms of molecular pathways, oncogenic ones, such as STAT3, NF-κB, and PI3K, that are involved in cancer growth and metastasis, are suppressed upon chrysin administration. MiRs are also potential targets of chrysin in cancer therapy that their expression is regulated. Noteworthy, since studies have shown that chrysin is capable of modulating the expression of miRs, further studies can focus on evaluating the effect of chrysin on other types of non-coding RNAs, such as long non-coding RNAs (lncRNAs) and circular RNAs (circRNAs).

Another potential application of chrysin is in suppressing chemoresistance. One of the major challenges in the field of chemotherapy is the resistance of cancer cells into the inhibitory effect of currently applied chemotherapeutic agents. Chrysin induces apoptosis to sensitize cancer cells into chemotherapy. Moreover, molecular pathways, such as Nrf2, that induce chemoresistance, are suppressed via chrysin. Further studies can focus on revealing other molecular pathways, such as miRs in chemoresistance, and the role of chrysin in their regulation.

In fact, different aspects of cancer cells are affected by chrysin, including proliferation, metastasis, and chemoresistance. These inhibitory effects are mediated via affecting both molecular pathways and mechanisms that were comprehensively discussed in the main text. As poor bioavailability is one of the drawbacks of chrysin in cancer therapy, a section was allotted to examine the role of nanoparticles for promoting bioavailability and the therapeutic effects of chrysin in cancer therapy. It is worth mentioning that these results were based on in vitro and in vivo experiments. Further studies can focus on evaluating the role of chrysin in clinical studies, which is important for clinical translation of chrysin.

## Figures and Tables

**Table 1:** Various pharmacological activities of chrysin in treating diseases.

Therapeutic Effect/Disease	In Vitro/In Vivo	Cell Line/Animal Model	Dose (In Vivo)/Concentration (In Vitro)	Duration of Experiment	Administration Route	Outcomes	Refs
Anti-hypertension	In vivo	Rat	100 mg/kg	18 weeks	Oral administration	Decreasing systolic and diastolic pressures Reducing insulin, angiotensin II and tiacylglycerols levels	[85]
Neuroprotective	In vivo	Rat	10 and 30 mg/kg	8 weeks	Oral gavage	Improving memory impairment Enhancing neuronal cell survival Reducing hippocampal neurogenesis depletion	[86]
Neuroprotective	In vivo	Rat	10, 30, and 100 mg/kg	3 weeks	Oral administration	Enhancing GPx activity and number of surviving cells in hippocampus Reducing MDA, NO and PGE2 levels Improving passive avoidance memory	[87]
Cardioprotective	In vitro	Cardiomyocyte	10, 50, and 100 µM	3 h	-	Decreasing aluminium-phosphide-mediated oxidative stress Reducing mitochondrial damage Improving mitochondrial function	[88]
Renoprotective Hepatoprotective	In vivo	Rat	100 mg/kg	-	-	Reinforcing antioxidant defense system via up-regulating GSH and SOD activities Reducing lipid peroxidation Decreasing inflammation via TNF-α down-regulation	[89]
Renoprotective Hepatoprotective	In vivo	Rat	25 and 50 mg/kg	7 days	Oral administration	Reducing AST, ALT, ALP, urea, creatinine, MDA and hepatorenal deterioration Enhancing SOD, CAT, and GPx activities Apoptosis inhibition via Bcl-2 up-regulation and Bax down-regulation Reducing inflammation via NF-κB down-regulation	[90]
Anti-diabetic	In vitro	Chorioretinal endothelial cells	1, 3, 10, 30, and 50 µM	24 h	-	Reducing Akt, ERK, MMP-2, and VEGF expressions	[91]
Anti-diabetic	In vivo	Rat model of type I diabetes	50 and 100 mg/kg	28 days	Oral gavage	Reducing oxidative stress index Enhancing glutathione levels	[92]
Gastric healing	In vivo	Mouse model of gastric ulcer via ethanol	10, 50, and 100 mg/kg	7 and 14 days	Oral administration	Apoptosis inhibition via caspase-3 down-regulation Reducing macroscopic lesions Enhancing catalase activity Improving inflammation via COX-2 down-regulation	[93]

**Table 2:** Chrysin administration in treating various cancers.

Cancer Type	In Vitro/In Vivo	Cell Line/Animal Model	Dose (In Vivo)/Concentration (In Vitro)	Period of Experiment	Administration Route	Outcomes	Refs
Prostate cancer	In vitro	DU145 and PC-3 cell lines	12.5, 25 and 50 µM	-	-	Induction of mitochondrion- and ER-mediated apoptosis Cell cycle arrest Down-regulation of MAPK and PI3K/Akt signaling pathways Impairing proliferation of PC cells	[144]
Gastric cancer	In vitro	MKN45 cells Mouse model of GC (created by CRIPSR/Cas9)	10, 20, 40, 80 and 160 µM 20 mg/kg	12, 24 and 45 h 14 days	Oral gavage	Suppressing migration Apoptosis induction Enhancing TET1 expression	[165]
Lung cancer	In vitro	A549 cells	2 and 5 µM	4 h	-	Down-regulation of MyD88 and TLR4 Inhibition of inflammation via NF-κB down-regulation Suppressing survival and metastasis	[134]
Cervical cancer	In vitro	HeLa cells	5, 10, 20 and 40 µM	0.5, 3, 6, 12 and 24 h	-	Down-regulation of NF-κB signaling pathway Inhibition of Twist/EMT axis Suppressing metastasis of cervical cancer	[182]
Breast cancer	In vitro	T47D breast cancer cells	20, 40, 60, 80, 100 and 120 µM	48 h	-	Disrupting proliferation of cancer cells via down-regulation of cyclin D1 and hTERT	[105]
Hepatocellular carcinoma	In vitro In vivo	Normal human hepatic cell LO2 and HepG2, Hep3B, Huh-7, HCC-LM3, Bel-7402 and SMMC-7721 Tumor xenografts	15, 30, and 60 µM 30 mg/kg	24, 48 and 72 h	Intraperitoneal injection	Down-regulation of HK-2 Suppressing glycolysis Apoptosis induction	[194]
Breast cancer Cervical cancer	In vitro	HeLa cells MCF-7 cells	15, 20, 25 and 30 µM	30 min	-	Significant reduction in survival of cancer cells Inducing both intrinsic and extrinsic apoptotic pathways P53-dependent apoptosis	[246]
Ovarian cancer	In vitro	SKOV3 cell line	5, 10 and 20 µmol/L	-	-	Decreasing the viability of cancer cells in a dose-dependent manner Down-regulation of CK2α, CD133 and CD44 Suppressing sphere formation capability	[247]
Breast cancer	In vitro	MDA-MB-231	10 µM	24 and 48 h	-	Inhibition of EGFR Reducing migration, growth and sphere formation ability of cancer cells	[109]
Breast cancer	In vitro In vivo	4T1 mouse breast cancer cells Balb/c mice implanted with 4T1 cells	60–100 µM 250 mg/kg	30 min 18 days	Oral administration	Suppressing lung metastasis Down-regulation of VEGF, and STAT3 Inhibiting proliferation	[119]
Prostate cancer	In vitro	Human prostate cancer cell line PC-3	10, 20, 30, and 40 µM	24, 48 and 72 h	-	Reducing the viability of cancer cells in a time- and dose-dependent manner Apoptosis induction	[248]
Cervical cancer	In vitro	Human cervical epidermoid carcinoma cell line ME180, and human cervical carcinoma cell lines HeLa, BU25TK− and SiHa	0–160 mg/mL	-	-	Apoptosis induction via caspase-3, caspase-9, and Bax up-regulation Stimulating mitochondrial dysfunction Cell cycle arrest induction	[184]
Liver cancer	In vitro	Hepatocellular carcinoma cells	5–100 µM	15, 30, 45 and 60 min	-	Mitochondrial dysfunction Cytochrome c release into the cytoplasm Apoptosis induction	[195]
Breast cancer	In vitro	MDA-MB-231 and MCF-7 cells	3–12 µM	-	-	Reducing the viability of cancer cells Apoptosis induction via capase-3 and caspase-7 up-regulation	[249]
Melanoma	In vitro In vivo	B16F10 cells Melanoma-bearing mice	12.5, 25, 50, and 100 µM 50 mg/kg	24 and 48 h 21 days	-	Induction of cell cycle arrest at G2/m phase Reducing tumor growth in vivo Promoting the anti-tumor activity of immune cells, such as macrophages and natural killer cells	[211]
Oral squamous cell carcinoma	In vitro	Oral squamous carcinoma KB cell line	1, 2, 4, 8, 16, and 32 µmol/L	24 h	-	Suppressing proliferation in a dose-dependent manner Apoptosis induction via capase-3 and caspase-7 up-regulation Inducing mitochondrial dysfunction Reducing the viability via down-regulation of PI3K/Akt signaling pathways	[250]
Bladder cancer	In vitro	Human bladder cancer cell lines T-24 and 5637 and the non-malignant immortalized urothelial SV-HUC-1 cells	20, 40 and 80 µM	24 h	-	Induction of ER stress via UPR activation Stimulating intrinsic pathway of apoptosis via caspase-3 and caspase-9 up-regulation Inhibition of STAT3 signaling pathway	[251]
Melanoma	In vitro	Human melanoma A375.S2 cell line	5, 10 and 15 µM	24 and 48 h	-	Impairing metastasis via VEGF, MMP-2, and N-cadherin down-regulation Enhancing E-cadherin expression Down-regulation of PI3K/Akt and NF-κB pathways in suppressing cancer proliferation	[182]
Colorectal cancer	In vitro	SW48, SW480, and SW620 CRC cells	5–50 µM	24 h	-	Enhancing ROS generation mTOR down-regulation Elevating LC-3II levels Autophagy induction Impairing cancer cell viability	[227]
Breast cancer	In vitro	MCF-7 cells	20 and 30 µM	48 and 72 h	-	Anti-proliferative activity in a dose- and time-dependent manner Apoptosis induction	[252]
Cervical cancer	In vitro	HeLa cells	0–10 µM	12–48 h	-	Stimulating apoptosis and cell cycle arrest Down-regulation of COX-2 expression	[253]
Colon cancer	In vitro	HT-29 cells	12.5, 25, 50, and 100 µg/mL	-	-	Induction of apoptosis via mitochondrial dysfunction Irradiation combined with chrysin exerts a synergistic effect	[235]
Thyroid carcinoma	In vitro In vivo	HTh7 and KAT18 cells	25, 50, and 75 µM 75 mg/kg	2–6 days 21 days	Oral gavage	Reducing the viability and growth via up-regulation of Notch1 and its down-stream target, Hes1	[254]
Hepatocellular carcinoma	In vitro	SMMC-7721 cells	10, 20 and 40 µM	24 and 48 h	-	Reducing sphere formation via STAT3 down-regulation	[203]
Breast cancer	In vitro	MCF-7 cells	40 µM	8 h	-	Decreasing cell viability by p53 activation through ATM-ChK2 axis Lack of DNA damage	[255]
Tongue squamous cell carcinoma	In vitro	CAL-27 cells	5, 25, 55 and 80 µM	24 h	-	Apoptosis induction via caspase-3 and caspase-9 up-regulation	[256]
Choriocarcinoma cells	In vitro	JAR and JEG3 cells	0–100 µM	24 h	-	Suppressing cell viability in a dose-dependent manner Inducing cell death via promoting ROS production and changing mitochondrial membrane potential	[257]
Colorectal cancer	In vitro	HCT116 cells	20, 30, 40 and 50 µM	36 h	-	Cell cycle arrest Migration inhibition PARPα up-regulation CYP2S1 and CYP1B1 induction	[245]
Colon cancer	In vitro In vivo	CT26 cells Allograft colon carcinoma model	10–200 µg/mL 0–10 mg/kg	24 and 48 h 28 days	Oral administration	Reducing tumor growth Induction of apoptosis via caspase-3 and caspase-9 up-regulation	[258]

**Table 3:** Chrysin-loaded nanoparticles in cancer therapy.

Nanovehicle	Cancer Type	In Vitro/In Vivo	Cell Line/Animal Model	Particle Size (nm)	Zeta Potential (mV)	Encapsulation Efficiency (%)	Outcomes	Refs
Micelle	Colorectal cancer	In vitro	Human-derived epithelial colorectal cancer cell lines HT-29	72–142	+10.1	77 (Docetaxel) 44 (chrysin)	Enhanced cellular uptake Effective inhibition of cancer stem cell migration	[292]
Polymeric micelles	Breast cancer	In vitro	MCF-7 cells	55	−2.7	87.6 (methotrexate) 86.5 (chrysin)	Enhancing efficacy of chrysin and methotrexate in breast cancer therapy via promoting cellular uptake	[293]
Dendrimer	Ovarian cancer	In vitro	Serous carcinoma (OSC) cell lines (OVCAR3 HTB-161™ and OVCAR8 CVCL_1629™) and a clear cell carcinoma (OCCC) cell line (ES2 CRL-1978™)	-	-	-	Selective targeting of cancer cells by folate functionalization of dendrimers High cellular uptake Remarkable decrease in survival of cancer cells	[297]
Polymeric nanoparticles	Breast cancer	In vitro	T47D breast cancer cell line	75	-	99.89	Higher cytotoxicity against breast cancer cells compared to chrysin alone	[304]
PLGA-PEG nanoparticles	Breast cancer	In vitro	T47-D breast cancer cell line	20–75	-	70	High cytotoxicity Excellent cellular uptake and encapsulation efficiency	[317]
PLGA-PEG nanoparticles	Colorectal cancer	In vitro	SW480 cells	50–140 nm	-		Higher cytotoxicity compared to chrysin and curcumin alone hTERT down-regulation	[307]
PLGA-PEG nanoparticles	Melanoma	In vivo	C57B16 mice bearing B16F10 melanoma tumours	285	−3.7	78.27 (curcumin) 83.5 (chrysin)	Enhancing expression of TIMP-1 and TIMP-2 Down-regulation of MMP-2 and MMP-9 Suppressing metastasis of cancer cells	[310]
Solid lipid nanoparticles	Breast cancer	In vitro	MCF-7 cells	Below 500	−20 to −47	More than 90%	High stability and promoting the anti-tumor activity of chrysin	[312]
PLGA-PEG nanoparticles	Breast cancer	In vitro	T47D cells	70–300	-	99.89	Accumulation in breast cancer cells High cytotoxicity	[318]
PLGA-PEG nanoparticles	Breast cancer	In vitro	MDA-MB-231 cells	305	−3.8	80.22 (curcumin) 85.25 (chrysin)	Synergistic effect Cell cycle arrest at G2/M phase Apoptosis induction Up-regulation of miR-132 and miR-502c	[319]
Copolymer nanoparticle	Lung cancer	In vitro In vivo	A549 cells Mice bearing an A549-derived tumor	77	−2.22	46.96	Enhanced cytotoxicity More potential in exerting tumor growth delay	[320]
Micelle	Breast cancer	In vitro	MCF-7 cells	152–420	−21.6	52–89	Promoting bioavailability of chrysin Exerting a 5-fold increase in anti-tumor activity	[321]
PLGA-PEG nanoparticles	Gastric cancer	In vitro	AGS cells	70–300	-	98.6	Decreasing cell survival via down-regulation of miR-18a, miR-21, and miR-221	[322]

## Abbreviations

ER	endoplasmic reticulum
ROS	reactive oxygen species
EMT	epithelial-to-mesenchymal transition
NAFLD	non-alcoholic fatty liver disease
TNF-α	tumor necrosis factor-α
IL	interleukin
I/R	ischemic/reperfusion
PD	Parkinson’s disease
TBI	traumatic brain injury
Nrf2	nuclear factor erythroid 2-related factor 2
EGFR	epidermal growth factor receptor
VEGF	vascular endothelial growth factor
HIF-1	hypoxia-inducible factor-1
STAT3	signal transducer and activator of transcription 3
TLRs	toll-like receptors
NF-κB	nuclear factor-kappaB
PC	prostate cancer
UPR	unfolded protein response
PERK	PRKR-like ER kinase
elF2α	eukaryotic translation initiation factor 2α
GRP78	78 kDa glucose-regulated protein
OC	ovarian cancer
GC	gastric cancer
5mC	5-methylcytosine
5hmC	5-hydroxymethylcytosine
miR	microRNA
TGF-β	transforming growth factor-beta
HK	hexokinase
HCC	hepatocellular carcinoma
ECM	extracellular matrix
5-FU	5-Fluorouracil
CRC	colorectal cancer
LC-3II	light chain-3II
mTOR	mammalian target of rapamycin
PPARα	Peroxisome proliferator-activated receptor alpha
CYP	cytochrome C
DTX	docetaxel
FDA	Food and Drug Administration
TIMPs	tissue inhibitors of metalloproteinases
lncRNAs	long non-coding RNAs
circRNAs	circular RNAs
